# F37. CACNA1C HYPOFUNCTION IN KETAMINE-ACTIVATED BRAIN NETWORKS IMPAIRS MEMORY CONSOLIDATION AND FUNCTIONAL BRAIN NETWORK CONNECTIVITY

**DOI:** 10.1093/schbul/sby017.568

**Published:** 2018-04-01

**Authors:** Rebecca Hughes, Cosima Willi, Jayde Whittingham-Dowd, Susan Broughton, Greg Bristow, Neil Dawson

**Affiliations:** Lancaster University

## Abstract

**Background:**

The CACNA1C gene, encoding the pore forming subunit of the calcium channel Cav1.2, is an established risk factor for a variety of psychiatric disorders including schizophrenia. The mechanisms through which altered Cav1.2 function increases the risk of developing these disorders is not well understood. Administration of subanaesthetic doses of the NMDA receptor antagonist ketamine provides a translational model of schizophrenia, modifying neuronal activity in brain networks underlying the symptoms of the disorder.

**Methods:**

Here we selectively reduce Cav1.2 expression in the brain networks activated by ketamine, using a novel transgenic mouse model (Cav-KHypo mice), and elucidate how this impacts on learning and memory, brain metabolism and functional brain network connectivity.

**Results:**

We show that the induction of Cav1.2 hypofunction in schizophrenia-relevant (ketamine-activated) brain networks impairs memory consolidation. In addition, Cav-KHypo mice show schizophrenia-relevant alterations in cerebral metabolism including prefrontal cortex, thalamic and hippocampal hypometabolism. The induction of Cav1.2 hypofunction in ketamine activated brain networks impairs memory consolidation by altering functional connectivity between neural systems underlying this process, including compromised prefrontal-hippocampal and compromised thalamic connectivity.

**Discussion:**

The data suggest that reducing CACNA1C expression in brain areas linked to schizophrenia is sufficient to induce disease related neural dysfunction. Furthermore, the data suggest that the regulation of neural system functional connectivity and memory consolidation may be primary mechanisms through which CACNA1C risk genes impact on cognition and increases the risk of developing psychiatric disorders.

